# Research on the Formation Characteristics of the Shaped Charge Jet from the Shaped Charge with a Trapezoid Cross-Section

**DOI:** 10.3390/ma15238663

**Published:** 2022-12-05

**Authors:** Bin Ma, Zhengxiang Huang, Yongzhong Wu, Yuting Wang, Xin Jia, Guangyue Gao

**Affiliations:** 1 School of Mechanical Engineering, Nanjing University of Science and Technology, Nanjing 210094, China; 2 China Ordnance Industry Navigation and Control Technology Research Institute, Beijing 100089, China; 3 Shanxi Jiangyang Chemical Co., Ltd., Taiyuan 030041, China

**Keywords:** shaped charge, trapezoid cross-section, formation, X-ray

## Abstract

The formation characteristics of the shaped charge jet (SCJ) from the shaped charge with a trapezoid cross-section is analyzed in this work. A theoretical model was developed to analyze the collapsing mechanism of the liner driven by the charge with a trapezoid cross-section. Based on the theoretical model, the axial and radial velocities of the SCJ from different trapezoid cross-section charges. The pressure model was employed to calculate the velocity for the subcaliber shaped charge, which was verified through numerical simulation. The results show that the influence of the angle of the trapezoidal charge (acute angle) on the axial velocity of the SCJ is not distinct, whereas the variation of the radial velocity of the shaped charge jet is obvious as the change in the angle of the trapezoidal charge. In addition, the related X-ray experiments were conducted to verify the theory. The theoretical results correlate with the experimental results reasonably well.

## 1. Introduction

As technology advances, many precision-guided weapons are emerging such as cruise projectiles, unmanned aerial vehicles, etc., [1] which are the basis of precision striking in the battlefield of the future. The structural characteristics of these precision-guided weapons present largely an asymmetrical cross-section in consideration of the aerodynamics [2]. Due to these structural constraints, the kinetic warhead and fragmentation warhead are captured extensively for these precision-guided weapons [3,4,5].

Shaped charge is one effective weapon against armored and concrete targets, and it has been given great attention around the world. It is well known that the shaped charge jet (SCJ) tip velocity can reach 6000–8000 m/s, even up to 10,000 m/s, and the tail element can fly a velocity of approximately 2000 m/s [6]. For the traditional axisymmetric shaped charge, the SCJ can be elongated considerably due to the existence of the strain rate ranging from 10^4^ to 10^5^ s^−1^ [7]. However, shaped charge is very sensitive to structural characteristics, and the asymmetry of the asymmetric section weapons is a challenge to the design of the shaped charge. Barry Stewart et al. [8] studied the feasibility of emerging a smear-compensated over-fly top attack SCJ. In their work, a novel side-mounted initiation train was designed to optimize space and maintain an antistructures emplacement capability. Experiments validated the initiation and two design variants and obtained a smear-compensated SCJ. Li Y-D et al. [9] studied the influence of an axially asymmetric shaped charge on the SCJ through a numerical simulation. Their conclusions showed that the detonation radius over the longitudinal axis—restricted by the charge radius over the same axis—as well as the detonation wave in the charge and the force acting on the liner, were affected, eventually influencing the jet velocity and shape. Wang Y et al. [10] obtained the morphology of the SCJ from shaped charges with square and circular cross-sections based on X-ray experiments. The results showed that the SCJ from the square cross-section shaped charge presented partially discrete phenomenon; they thought it could adjust the compaction rate of the SCJ from the square cross-section shaped charge through changing the inscribed circle diameter of the charge cross-section. In addition, Żochowski, Paweł et al. [11,12] researched and characterized the main parameters of the shaped charge jet formed and the penetration capability of the more contemporary high-hardness (500 HB) ARMSTAL 30PM steel armor based on a simulation and experiments. They showed that their numerical model of the warhead was defined more accurately than in previously published studies, since it was based on the real grenade dimensions and technical documentation.

While the influence of the asymmetry factors such as asymmetrical initiation, assembly error, etc., on the SCJ performance has been investigated extensively and some experiments related to the shaped charge with noncircular cross-section have been carried out, there is limited research exploring the formation characteristics of the SCJ from shaped charge with a trapezoid cross-section. Therefore, this paper aims to improve upon this work. Here, the X-ray experiments were carried out for the SCJ formation process of the shaped charge with two trapezoid cross-sections. A theoretical model for calculating the axial and radial velocities is then developed to describe the velocity characteristics of the SCJ.

## 2. Formation Characteristics of the SCJ from the Shaped Charge with a Trapezoid Cross-Section

### 2.1. Liner Collapsed Theory for the Shaped Charge with a Trapezoid Cross-Section

**Figure 1:**
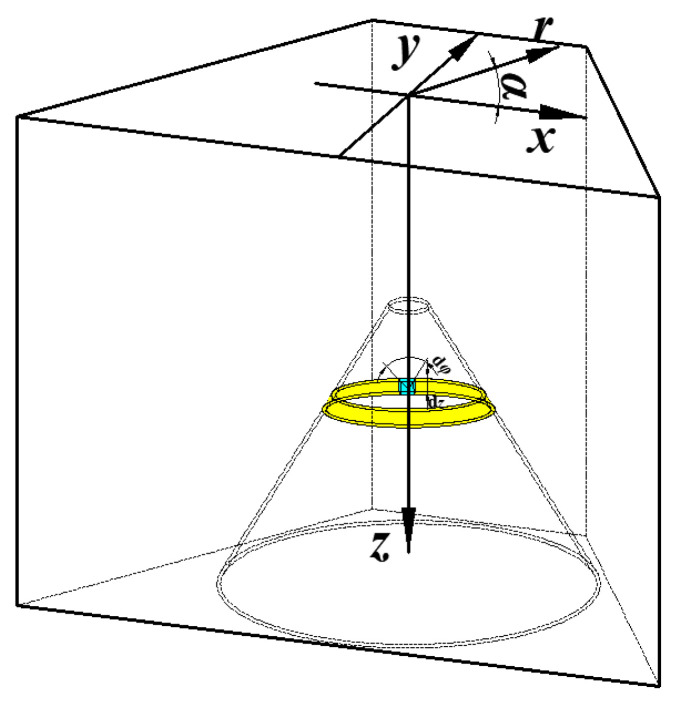
Three-dimensional system of coordinates in the shaped charge with a trapezoid cross-section.

The SCJ formation for the shaped charge with a trapezoid cross-section is not an axisymmetric 2-dimensional problem, but a complex 3-dimensional problem. To explore the SCJ formation characteristics of the shaped charge with a trapezoid cross-section, the coordinate system as shown in Figure 1 was established, in which the rectangular coordinate system and the cylindrical coordinate system are all involved. In Figure 1, the highlighting represents the infinitesimal of the liner taken as the research object.

**Figure 2:**
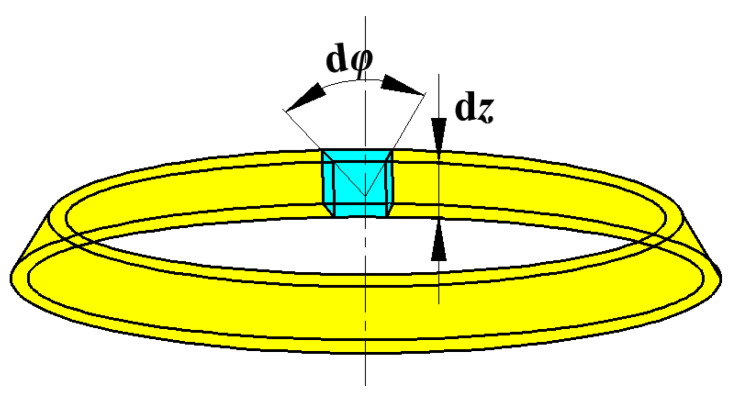
The infinitesimal element of the liner.

The infinitesimal of the liner for the study is shown individually in Figure 2. In this model, *dφ* and *dz* are the degree and the height along *z* axis. For the infinitesimal element, the mass can be expressed as:

(1) $$dm=\rho_{m}\pi \left(r_{e}{}^{2}-r_{i}{}^{2}\right)dz\frac{d\varphi }{2\pi }$$

where *ρ_m_* is the density of liner material, and *r_e_* and *r_i_* are the external radius and the inner radius, respectively.

The process of the explosive driving the liner can be divided into two phases based on the detonation products action process. In the first stage, the shaped charge is detonated, the detonation wave propagates in the explosive with a spherical wave front, and the detonation wave arrives at the surface of the liner at time *t*_0_. The state of the detonation product on the surface of the liner starts static to kinetic and is accompanied by the rarefaction wave. Furthermore, the infinitesimal element of the liner begins moving under the combined action of the detonation product and the rarefaction wave. In the second stage, the detonation wave propagates to the side of the charge, and a series of lateral rarefaction waves appear and diffuse to the detonation product. At time *t_b_*, the lateral rarefaction waves spread to the surface of the liner, in which the reflected waves are also formed on the surface of the liner. The liner is collapsed to form the SCJ under the comprehensive function of many factors.

During the research, the initial coordinate of the liner infinitesimal element was set as (*x*_0_, *y*_0_, *z*_0_), and *t*_0_ and can be written:

(2) $$t_{0}=\frac{\sqrt{x_{0}{}^{2}+y_{0}{}^{2}+z_{0}{}^{2}}}{D_{J}}$$

where *D_J_* is the detonation velocity of the explosive. 

When the detonation wave arrives at the surface of the liner, the initial pressure on the surface of the liner is the C-J pressure of the explosive and can be obtained as follows [13,14]:

(3) $$p_{0}=\frac{1}{k+1}\rho_{0}D_{J}{}^{2}$$

where *k* and *ρ*_0_ are the adiabatic exponent and the initial density of the explosive, respectively.

The initial state of the detonation products on the surface of the liner can be described as [15]:

(4) $$\left\{\begin{array}{l} u_{0}=0\\ c_{0}=\frac{1}{2}D_{J} \end{array}\right.$$

where *u*_0_ is the initial velocity of the detonation product particle and *c*_0_ is the sound velocity of the detonation product.

This assumes that the motion of the detonation product on the surface of the liner is an isentropic process [16] and *γ* is the isentropic exponent. The pressure of the detonation product on the surface of the liner can be derived as follows:

(5) $$p(t)=p_{0}\cdot {\left(\frac{c}{c_{0}}\right)}^{\frac{2\gamma }{\gamma -1}}$$

where *c* is the sound velocity of the detonation product on the surface of the liner at time *t*.

Furthermore, the motion equation of the liner infinitesimal element can be linked to the pressure of the detonation product on the basis of Newton’s Second Law, in which it can be given as:

(6) $$dm\frac{dV}{dt}=p(t)ds$$

where *ds* is the area of the liner infinitesimal element contacting with the detonation product and *V* is the velocity of the liner infinitesimal element.

### 2.2. Calculation Model for the State Parameters of the Detonation Product on the Liner Surface

The shaped charge with a trapezoid cross-section has a 3-dimensional structure, and the profile of the shaped charge was selected as the research object to carry out the theoretical analysis. In the process of the liner being collapsed, the rarefaction wave is a critical factor.

**Figure 3:**
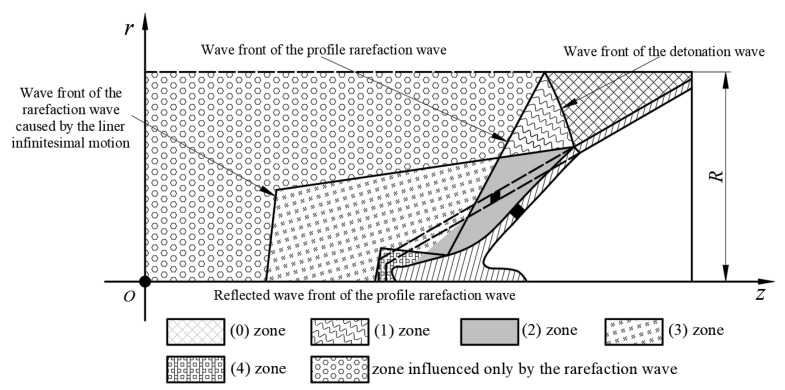
Distribution of the detonation product that is not influenced by the rarefaction wave.

When the detonation product is not influenced by the rarefaction wave, the distribution of the detonation product can be expressed as in Figure 3. 

The state of the detonation product on the surface of the liner can be rewritten as:

(7) $$\left\{\begin{array}{l} u-\frac{2}{\gamma -1}c=-\frac{2}{\gamma -1}c_{0}\\ x=(u+c)(t-t_{0})+F_{2}(u,c) \end{array}\right.$$

where *u* is the velocity of the detonation product particle and *F*_2_(*u*, *c*) is a function related to the motion characteristic of the liner infinitesimal element.

Considering that the velocity of the detonation product particle on the surface of the liner is equal to the motion velocity of the corresponding liner infinitesimal element, it can be derived as follows:

(8) $$\frac{dc}{dt}=\frac{\gamma -1}{2}\frac{ds}{dm}p_{0}c_{0}^{\frac{2\gamma }{\gamma -1}}c^{\frac{2\gamma }{\gamma -1}}$$

Therefore, the sound velocity of the detonation product is obtained from the time integration of Equation (8):

(9) $$c={\left(\frac{\gamma +1}{2}\frac{ds}{dm}p_{0}c_{0}^{\frac{2\gamma }{\gamma -1}}(t-t_{s})+c_{0}^{\frac{1+\gamma }{1-\gamma }}\right)}^{\frac{1-\gamma }{1+\gamma }}$$

where *t_s_* is the time of the rarefaction wave entering the detonation product.

**Figure 4:**
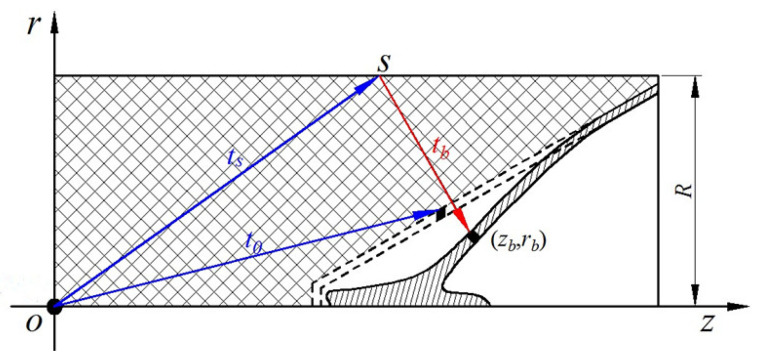
Calculation for the state-transforming moment of the detonation product.

The detonation wave propagates to the profile of the charge, and the rarefaction wave is formed to start to spread into the detonation product. The appearance of the rarefaction wave influences the collapsing process of the liner. The diagram of the arising moment of the rarefaction wave is shown in Figure 4. 

The shortest path of the profile of the rarefaction wave arriving at the liner infinitesimal element is perpendicular to the generatrix of the liner (red line in Figure 4) and the profile rarefaction wave enters from *s* at time *t_s_*. This rarefaction wave arrives to the surface of the liner at time *t_b_*. Based on the geometrical relationship from Figure 4, the coordinates of the liner infinitesimal element at time *t_b_* can be calculated as:

(10) $$\left\{\begin{array}{l} z_{b}=z_{0}+\int_{t_{0}}^{t_{b}}V(t)\sin(\alpha +\delta (t))dt\\ r_{b}=r_{0}-\int_{t_{0}}^{t_{b}}V(t)\cos(\alpha +\delta (t))dt \end{array}\right.$$

where 2*α* is the cone angle of the liner, (*z*_0_, *r*_0_) is the initial coordinate of the liner infinitesimal element in *roz* coordinate system, and *V*(*t*) and *δ*(*t*) are the velocity and direction angle of the liner infinitesimal element at time *t*, respectively.

After derivation, *t_b_* can be expressed as:

(11) $$t_{b}=\frac{\sqrt{{\left[z_{b}-(\sqrt{R-r_{b}})\tan\alpha \right]}^{2}+R^{2}}}{D_{J}}+\frac{R-r_{b}}{c_{s}\cos\alpha }$$

where *R* is the charge radius in section and *c_s_* is the velocity of the rarefaction wave. This can be also written as:

(12) $$c_{s}=\frac{k}{k+1}D_{J}$$

**Figure 5:**
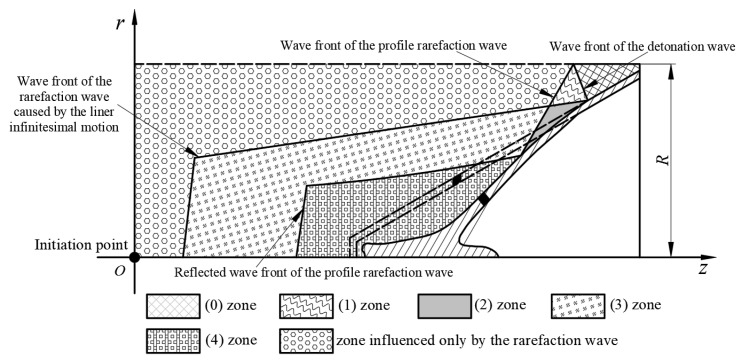
Distribution of the detonation product influenced by the rarefaction wave.

Due to the emergence of the rarefaction wave, the collapsing process of the liner is influenced. The distribution of the detonation product with the influence of the rarefaction wave is shown in Figure 5.

The state of the detonation product under the action of the rarefaction wave can be described as:

(13) $$\left\{\begin{array}{l} x=(u-c)(t-t_{s})+x_{s}\\ x=(u+c)(t-t_{0})+F_{4}(u,c) \end{array}\right.$$

where *x_s_* is the distance between the incoming point of the rarefaction wave and the initial position of the liner infinitesimal element, and *F*_4_(*u*, *c*) is a function related to the motion characteristic of the liner infinitesimal element. 

Combining $\frac{dx}{dt}=u=V$, the sound velocity of the detonation product on the liner surface can be derived as follows:

(14) $$c={\left(E{\left(t-t_{s}\right)}^{\frac{\gamma +1}{\gamma -1}}-\frac{\gamma +1}{2}\frac{ds}{dm}p_{0}c_{0}^{\frac{2\gamma }{\gamma -1}}(t-t_{s})\right)}^{\frac{1-\gamma }{1+\gamma }}$$

where *E* is the integration constant, which depends on the parameters of the detonation product from II zone. 

### 2.3. Velocity Analysis of the SCJ from the Shaped Charge with a Trapezoid Cross-Section

**Figure 6:**
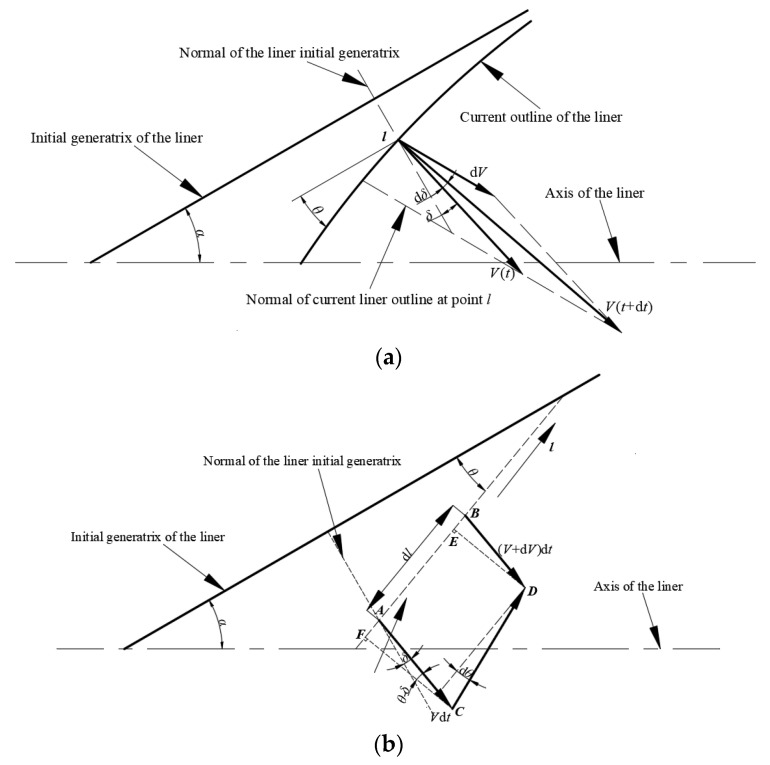
Velocity vector of the liner infinitesimal element. (**a**) Velocity vector of the liner infinitesimal element; (**b**) Relationship between deflection angle and velocity for the liner infinitesimal element.

Chou [17] indicated that the collapsing velocity of the liner along its generatrix was variational, as is shown in Figure 6a. In this figure, *V* is the velocity value of the liner infinitesimal element, *δ* is the angle between the motion direction and the normal of the liner generatrix, and *θ* is the angle between the initial tangent and the current tangent on the liner surface. In time *dt*, the velocity vector of the liner infinitesimal element, $\vec{V}(t)$, has an increment $d\vec{V}$, and the velocity of the liner infinitesimal element is marked as $\vec{V}(t+dt)$ at time *t* + *dt*.

This assumes that the detonation product always acts vertically on the surface of the liner infinitesimal element, so the increment value of the velocity can be obtained as:

(15) $$dV=\left|d\vec{V}\right|\cos(\theta -\delta )$$

The relation between *θ* and the velocity of the liner infinitesimal element is shown in Figure 6b.

According to Figure 6, this can be obtained as:

(16) $$d\delta =\frac{\left|d\vec{V}\right|}{V}\sin\left(\theta -\delta \right)$$

In the process of the research, *θ*(*l*) was defined as the angle between the tangent line at A and the initial generatrix of the liner. According to the geometrical relationship shown in Figure 6b, this can be derived from:

(17) $$\frac{d\theta }{dt}=-\frac{dV}{dl}\cos\left(\theta -\delta \right)$$

The collapsing characteristics of the liner can be described through collapsing parameters of the liner infinitesimal element, such as collapsing velocity *V* and ejection angle *δ*.

Considering the pressure model, the expression can be derived as follows:

(18) $$\frac{\left|d\vec{V}\right|}{dt}=\frac{ds}{dm}p$$

This equation integrates Equation (15), and the velocity of the liner infinitesimal element at time *t* can be rewritten as:

(19) $$V=\int_{0}^{t}\frac{ds}{dm}p\cos(\theta -\delta )dt$$

Combining Equations (16) and (18), we can obtain the expression:

(20) $$\frac{d\delta }{dt}=\frac{ds}{dm}p\frac{\sin\left(\theta -\delta \right)}{V}$$

Based on the above analysis, we can calculate the parameters *V*, *δ*, and *θ*, through which the collapsing process of the liner can be further defined.

**Figure 7:**
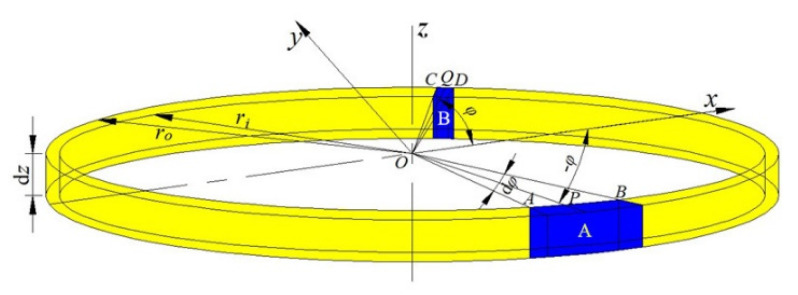
Symmetrically-distributed liner infinitesimal elements about the *roz* plane.

For the shaped charge with the trapezoid cross-section, the collapsing velocities of the liner infinitesimal elements at the same height of the liner are inconsistent. During the analysis, the *roz* plane is a symmetry plane in a coordinate system. As is shown in Figure 7, elements A and B are symmetrically-distributed liner infinitesimal elements about the *roz* plane. 

This takes the annular element as the research object, and *dM_j_* and *dM_s_* represent the mass of the SCJ and the slug from the element, respectively. Thus, the expression can be given as:

(21) $$\begin{array}{l} dM_{j}=\sum_{i=1}^{N}dm_{ji}\\ dM_{s}=\sum_{i=1}^{N}dm_{si} \end{array}$$

where *N* = π/*dφ* and *dφ* are the angles of the liner infinitesimal element, as shown in Figure 7.

Considering the law of the conservation of momentum [18], the axial and the radial velocities of the SCJ and the slug from the annular liner element can be derived as follows:

(22) $$\begin{array}{l} V_{jz}=\frac{\sum_{i=1}^{N}dm_{ji}v_{jzi}}{\sum_{i=1}^{N}dm_{ji}},\;V_{sz}=\frac{\sum_{i=1}^{N}dm_{si}v_{jsi}}{\sum_{i=1}^{N}dm_{si}}\\ V_{jx}=\frac{\sum_{i=1}^{N}2dmv_{xi}}{dM_{J}+\frac{V_{sz}}{V_{jz}}dM_{s}},\;V_{sx}=\frac{\sum_{i=1}^{N}2dmv_{xi}}{dM_{J}+\frac{V_{jz}}{V_{sz}}dM_{s}} \end{array}$$

### 2.4. Numerical Simulation

**Figure 8:**
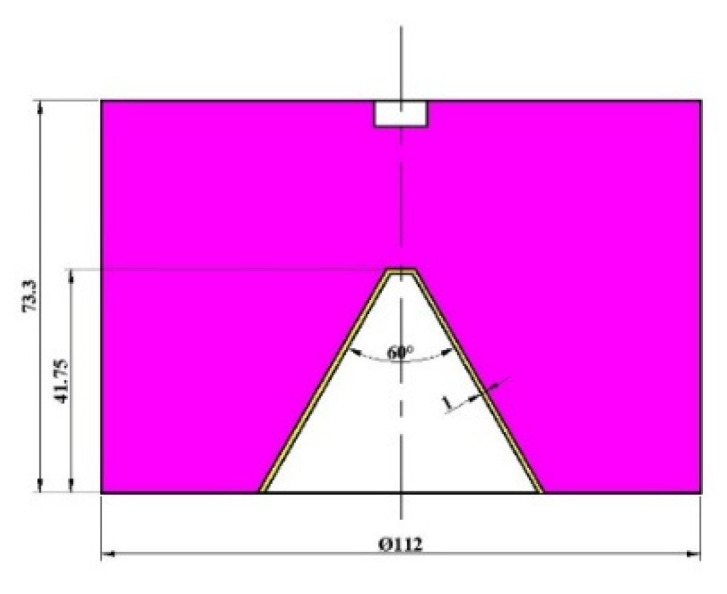
Structure of the Φ112 mm shaped charge.

In this work, the shaped charge with the trapezoid cross-section was different from the cylindrical-shaped charge, so the diameter of the liner smaller than the diameter of the charge (subcaliber) appears. During the simulation, the Φ112 mm shaped charge was carried out to verify the correctness of the pressure model for the subcaliber shaped charge. The structure of the Φ112 mm shaped charge is shown in Figure 8.

**Figure 9:**
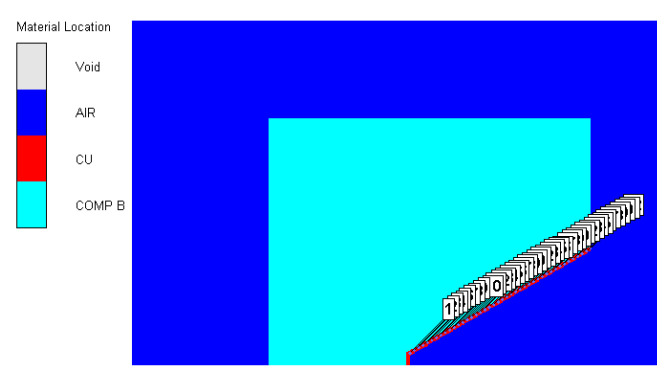
Simulation model of the Φ112 mm shaped charge.

According to the structure of the shaped charge from Figure 8, the simulation model was built through AUTODYN. In the model, the material of the liner is the oxygen-free high-conductivity copper (OFHC) and the charge is the CMOP B explosive. For obtaining the collapsing velocity in different liner infinitesimal elements, the Gaussian points (a total of 42) were set, as is shown in Figure 9.

**Figure 10:**
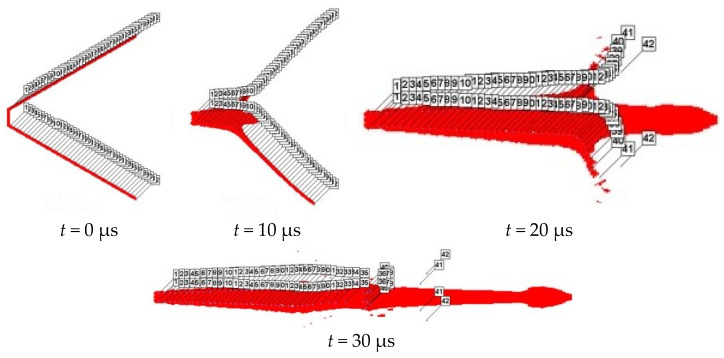
Liner collapsed process for the Φ112 mm shaped charge.

Based on the model built, the collapsing process of the liner at a typical moment can be acquired, as shown in Figure 10. These are the collapsing states of the liner at the initial moment, 10 μs, 20 μs and 30 μs after charge detonation. In the deformation process of the liner, the Gaussian points move as the material flows, and they can record the parameter variation of the liner infinitesimal elements. 

**Figure 11:**
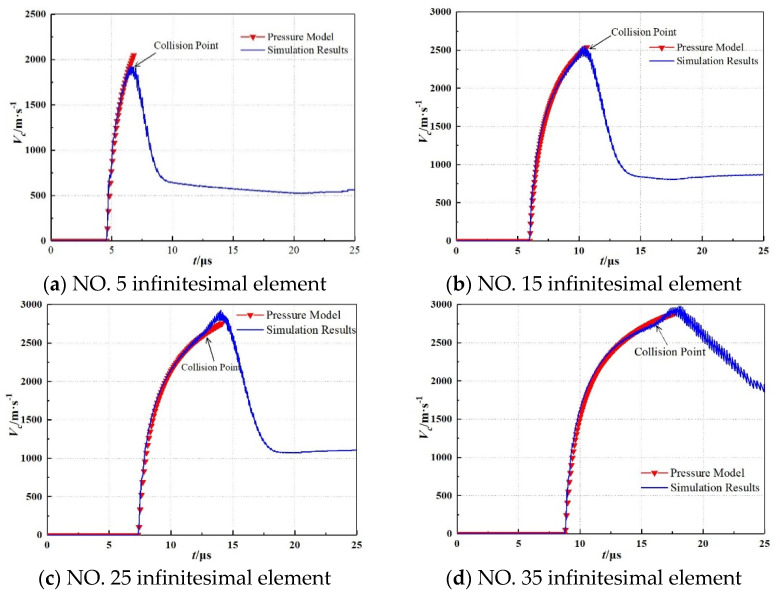
Acceleration process comparison between the calculation and the simulation for different liner infinitesimal elements.

According to the simulation results, the Gaussian points numbered as 5, 15, 25, and 35 were selected to capture the collapsing velocity of the corresponding infinitesimal element, in which the collapsing velocities from the simulation were compared with calculated results employed by the pressure model. These the results are shown in Figure 11, which indicates that the theoretical results correlated with the simulation results reasonably well. Therefore, the pressure model employed to calculate the velocity for the subcaliber shaped charge is feasible.

During the analysis, the liner infinitesimal elements numbered 5, 15, 25, and 35 were selected to compare the acceleration process from the theoretical calculation and the simulation. The infinitesimal elements selected reflect the overall collapsing process of the liner. As shown in Figure 11, the theoretical calculation adopting the pressure model was consistent with the simulation results for the collision velocity of the liner, which indicates that the pressure model for calculating the collapsing velocity of the liner is feasible. 

**Figure 12:**
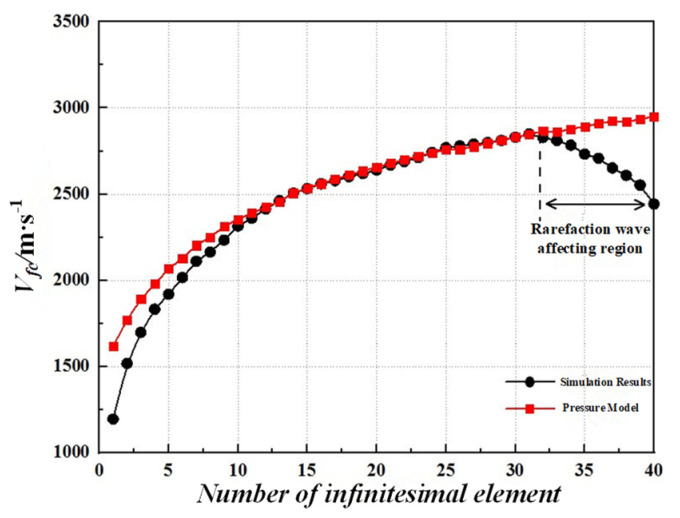
Ultimately collapsing velocity of liner infinitesimal elements from the pressure model and the simulation.

The ultimately collapsing velocities from the calculation and the simulation are shown in Figure 12, which indicates a reasonably good correlation, especially for the first 32 infinitesimal elements. In addition, some variation between the theoretical and the simulation results of the collapsing velocity appear after the NO. 32 infinitesimal element. The reasons for the discrepancies are that the influence of the rarefaction waves is not considered in the theoretical model, and the influence is more obvious near the liner bottom. In total, the ultimately collapsing velocities from the calculation are consistent with those from the simulation; the pressure model was employed to calculate the ultimately collapsing velocities of the liner, which proved to be reasonable. 

### 2.5. Results and Discussion

**Figure 13:**
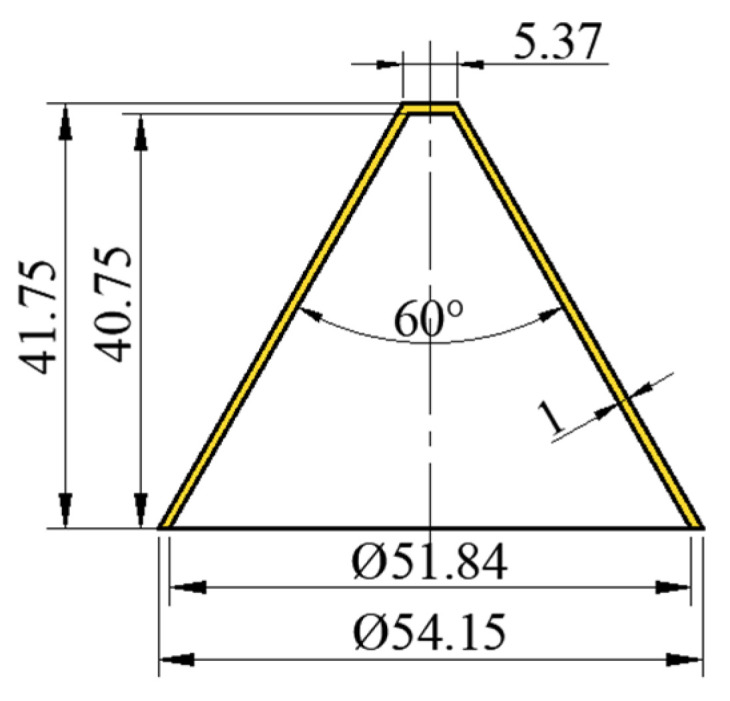
Structure of the liner used in this study.

During the research, the shaped charge with the isosceles trapezoid cross-section was analyzed and used to explore the influence of the non-axial symmetry of the charge on the formation characteristics of the SCJ. In the shaped charge with the isosceles trapezoid cross-section, the explosive employed was Comp. B and the material of the liner was oxygen-free high-conductivity copper (OFHC). The structure of the liner is shown in Figure 13. 

**Figure 14:**
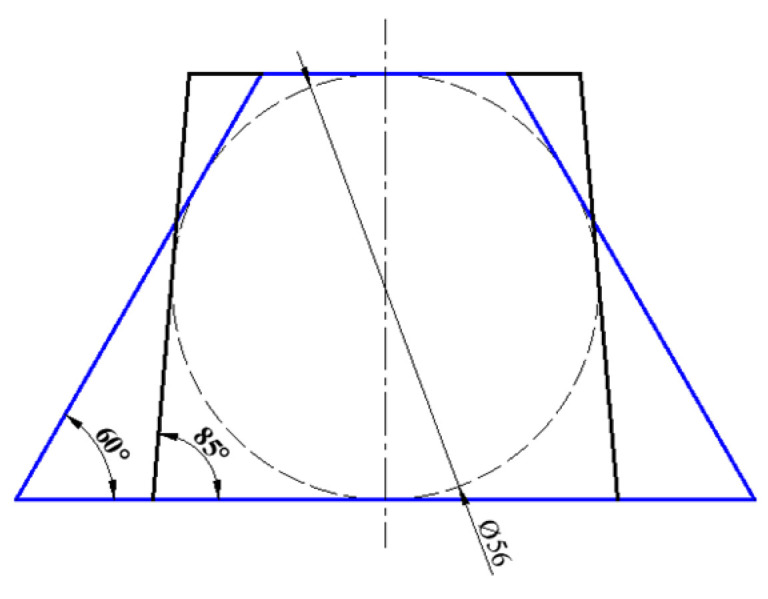
Planform of the shaped charge with different acute angles.

For the shaped charges used in the calculation, the cross-sections are shown in Figure 14. To research the influence of the variation of the acute angle (base angle of the trapezoid) on the SCJ formation, the structure of the shaped charges with the different base angles of the trapezoid was calculated. 

For the shaped charge, the inscribed circle diameter of the trapezoid cross-section is 56 mm, and the height of the charge is 73.3 mm. 

**Figure 15:**
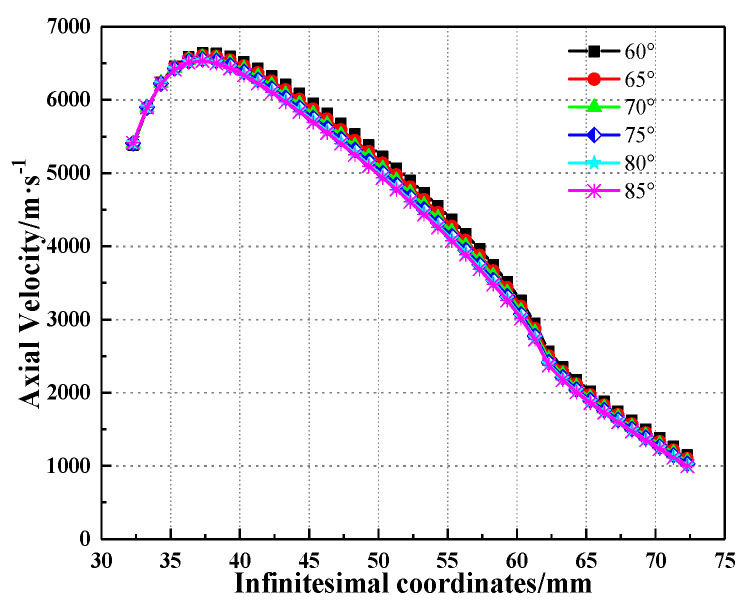
Variation of the SCJ axial velocity with infinitesimal coordinates under different base angles of the trapezoid.

Based on the theoretical model established in this work, the axial velocity of the SCJ with different base angles of the trapezoid was calculated, and the result is shown in Figure 15. According to the axial velocity of the shaped charge jet formed by different liner infinitesimal elements, the distance between the original position on the liner and cone axis is not large enough for the elements to accelerate to their final collapse velocity in the region near the cone apex of the liner, and the inverse velocity gradient of the SCJ elements from the apex end of the liner leads to the accumulation in SCJ tip. During the calculation, the acute angle of the trapezoid increased in intervals of 5° from 60° to 85°, and the SCJ axial velocity changed from 6636 to 6533 m/s, which is not a significant change. 

**Figure 16:**
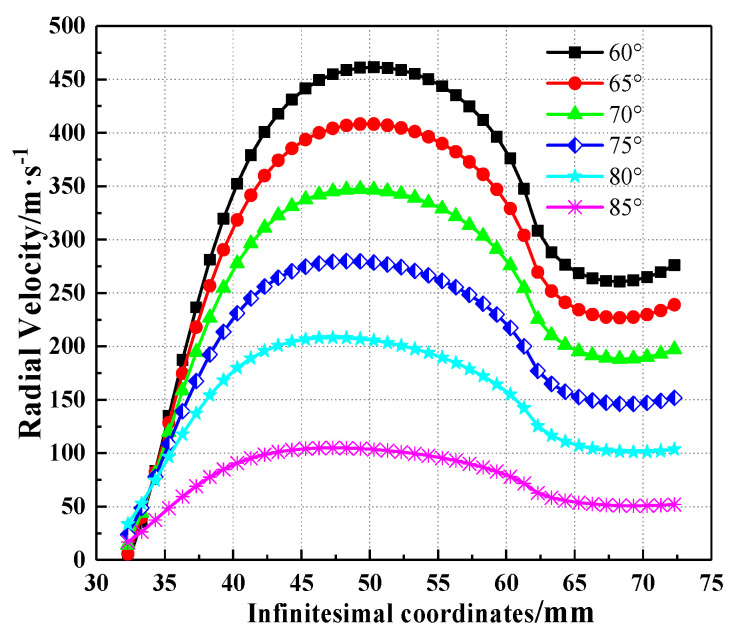
Variation of the SCJ radial velocity with infinitesimal coordinates under different base angles of the trapezoid.

The SCJ radial velocity is an important factor affecting the SCJ stability, so the SCJ radial velocities were calculated based on the theoretical model for different charge structures. The SCJ radial velocities were also obtained, as shown in Figure 16.

According to Figure 16, the variation of the SCJ radial velocity due to the effect of the variation of the base angle of the trapezoid is obvious. The maximum of the SCJ radial velocity was 461.6 m/s for the acute angle of the trapezoid being 60°, which decreased to 408.2, 347.3, 279.9, 208.3 and 105.0 m/s when the base angle increased by 5°from 65° to 85°, respectively. Figure 14 shows that as the base angle changed from 65° to 85°, the asymmetry of the charge gradually weakened, and the symmetry of the detonation waves produced by the detonation of the explosive enhanced gradually.

## 3. Experiments and Results

### 3.1. The Shaped Charge with a Trapezoid Cross-Section Used in the Experiments

**Figure 17:**
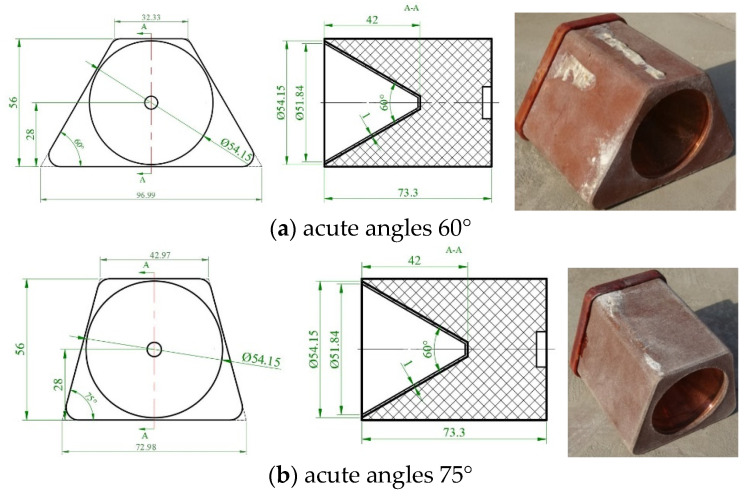
Shaped charge with a trapezoid cross-section.

The cross-section of the shaped charge used in the experiments was a trapezoid with a length of 73 mm. In the experiments, the SCJ formations of the shaped charge with the acute angles of 60° and 75° were filmed, in which the thickness of the conical copper liner was 0.8 mm and the cone angle was 60°. In addition, the explosive used was a Comp. B high explosive, and a fusion cast process was adopted. The structures and the physicals are shown in Figure 17.

**Figure 18:**
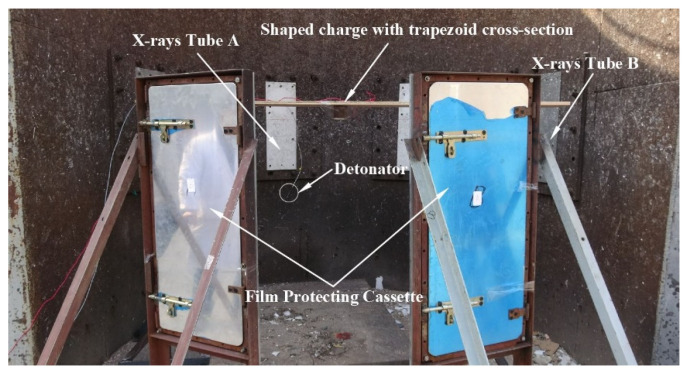
Experimental setup for the X-ray test.

Two 450 kV flash X-ray exposures were used to visualize the shape of the SCJ produced by the shaped charge with a trapezoid cross-section. The four films protected by a protective cassette were used to capture images of the SCJ. To calculate the SCJ velocity based on the data from the film, a mark was made in the film protected cassette, which can give a benchmark for the SCJ tip and tail at different times. The diagram of the experimental setup is shown in Figure 18. The exposure time of the flash X-ray in experiments was set as 30 and 40 μs after the jet tip arrived at the bottom of the liner for the shaped charge with the acute angles of 60°, and 40 and 50 μs for the shaped charge with the acute angle of 75°, respectively. 

### 3.2. Results and Discussion

**Figure 19:**
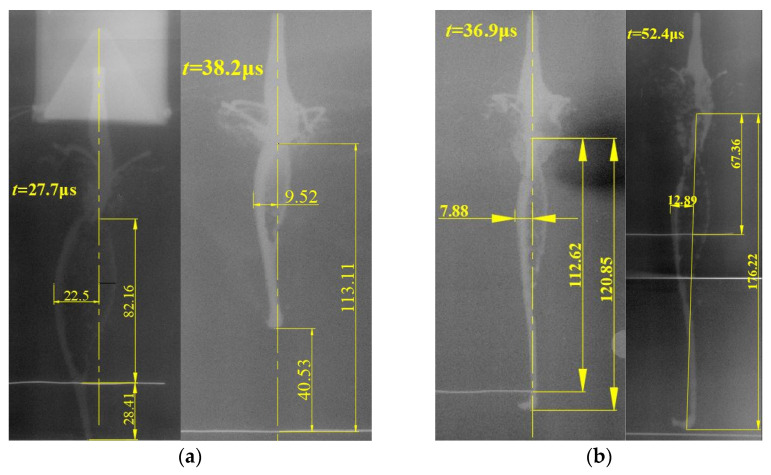
X-ray image of the SCJ from the shaped charge with a trapezoid cross-section. (**a**) SCJ produced by the shaped charge with an acute angle of 60°, (**b**) SCJ produced by the shaped charge with an acute angle of 75°.

To obtain the characteristics of the SCJ produced by the shaped charge with a trapezoid cross-section, the X-ray experiments were carried out. The flash X-ray radiographs of the SCJ are shown in Figure 19. In the process of the experiments, the bottom of the shaped charge was used as a reference point, and the moment of the detonation wave reaching the bottom of the shaped charge was time zero. For catching the morphological characteristics and kinetic parameters, the X-ray radiographs at 27.7 and 38.2 μs were finally captured for the SCJ produced by the shaped charge with cross-section acute angle of 60°, and were 36.9 and 52.4 μs for the cross-section acute angle of 75°. 

It should be noted that the variation between the setting exposure time and the real exposure time exists due to control precision of the X-ray system, in which the variations of the exposure time present no effect on the analysis of the experimental results. During the calculation of the related parameters, the real exposure time was used. The related measurement parameters based on the flash X-ray radiographs are shown in Table 1.

According to the X-ray radiographs, the axial and radial velocities were calculated. The axial velocities of the SCJs from the shaped charge with the acute angles of 60° and 75° are 6.57 and 6.49 m/s, and the theoretical values are 6636 and 6558 m/s, respectively. The errors between these values were 1% and 1.05%, respectively. In addition, the average radial velocities were 466.35 and 279.9 m/s for these two angles, and the theoretical values were 461.6 and 229.77 m/s, respectively. The errors between these values are 1.02% and 17.9%, respectively. The measurement error may contribute to the variation observed. Based on the outcome analysis, the theoretical results correlate with the experimental results reasonably well.

## 4. Conclusions

The formation characteristics of the SCJ from the shaped charge with a trapezoid cross-section was researched on the basis of the theory and X-ray experiments. Based on the research output presented above, the following conclusions may be drawn:

1. A theoretical model was employed to describe the formation characteristics of the SCJ from the shaped charge with a trapezoid cross-section. Based on the model, we analyzed the axial and radial velocities of the SCJs produced by the trapezoid cross-section shaped charge with different acute angles.
2. The acute angle of the trapezoid increased in intervals of 5° from 65° to 85°, and the SCJ axial velocity changed from 6636 to 6533 m/s, which is not a significant change.
3. The maximum of the SCJ radial velocity was 461.6 m/s for the acute angle of the trapezoid being 60°, which decreased to 408.2, 347.3, 279.9, 208.3 and 105.0 m/s when the base angle increased in intervals of 5° from 65° to 85°, respectively. This indicated that the asymmetry of the charge is gradually weakened as the acute angle increases, and the symmetry of the detonation waves produced by the detonation of the explosive is gradually enhanced.
4. The X-ray experiments were conducted to verify their theoretical validity. The results showed that the axial velocities of the SCJs from the shaped charge with the acute angles of 60° and 75° were 6570 and 6490 m/s, and the errors with the theoretical values were 1% and 1.05%, respectively. The average radial velocities were 466.35 and 279.9 m/s for those two cases, and the errors were 1.02% and 17.9%, respectively. This provides reasonable support for the current theory.

## Figures and Tables

**Table 1:** Experimental results.

No.	*t_e_*/μs	*Z_tip_*/mm	*Z_tail_*/mm	*R*/mm	*v*_tip_/m·s^−1^	*v*_tail_/m·s^−1^	*v_R_*/m·s^−1^	
1	27.7	40.53	113.11	9.52	6570	2948	343.68	466.35
2	38.2	−28.41	82.16	22.5			589.00	
1	36.9	−8.23	112.62	7.39	6490	2920	213.55	229.77
2	52.4	−108.91	67.36	12.89			245.99	

## Data Availability

Not applicable.
